# Health providers pass knowledge and abilities acquired by training in obstetric emergencies to their peers: the average treatment on the treated effect of PRONTO on delivery attendance in Mexico

**DOI:** 10.1186/s12884-018-1872-4

**Published:** 2018-06-15

**Authors:** Jimena Fritz, Héctor Lamadrid-Figueroa, Gustavo Angeles, Alejandra Montoya, Dilys Walker

**Affiliations:** 1 Division of Reproductive Health, Research Center for Population Health, National Institute of Public Health (INSP), Av. Universidad 655, Col. Santa María Ahuacatitlán, 62100 Cuernavaca, Morelos Mexico; 2 Department of Maternal and Child Health, University of North Carolina at Chapel Hill (UNC), Chapel Hill, North Carolina USA; 3 Department of Obstetrics, Gynecology and Reproductive Sciences, Bixby Center for Global Reproductive Health, University of California in San Francisco, (UCSF), San Francisco, California USA

**Keywords:** Program evaluation, Labor stage, First, Delivery, Obstetric, Mexico

## Abstract

**Background:**

A significant proportion of newborn and maternal deaths can be prevented through simple and cost-effective strategies. The main aim of this study was to evaluate the impact of the PRONTO obstetric-emergency management training for improving evidence-based birth attendance practices among providers attending the training at 12 hospitals in three states of Mexico from 2010 to 2012, and to estimate dissemination of the training within the hospitals.

**Methods:**

The average treatment on the treated effect of the PRONTO intervention for the probability of performing certain practices during birth attendance was estimated in a sample of 310 health providers. Impact estimates were obtained by performing provider-level matching using a mixed Mahalanobis distance one-to-one nearest-neighbor and exact matching approach. A secondary analysis estimated the positive externalities caused by the intervention in the treated hospitals using the same analytical approach. Provider-level fixed effects regression models were used to estimate the rate of decay of the probability of performing the examined practices.

**Results:**

Providers attending the PRONTO training showed significant increases in the probability of performing the complete active management of the third stage of labor, especially the first and third steps, and skin-to-skin-contact. There was a negative and significant effect on the probability of performing uterine sweeping. Providers who did not attend the training in treated hospitals also showed marked significant changes in the same practices, except for uterine sweeping. There was no evidence of a significant decay of the probability of performing the routine practices over time among the treated providers.

**Conclusions:**

PRONTO is efficacious in changing trained providers’ behavior, but not on all practices, suggesting that some practices are deeply ingrained. The results also suggest that information on practices is effectively transmitted to peers within treated hospitals. Previous findings of the dilution of the effect of PRONTO on some practices seem to be more related to the rotation of personnel (mainly interns) rather than providers returning to their former habits.

**Trial registration:**

NCT01477554. Registered on November 18, 2011; retrospectively registered.

**Electronic supplementary material:**

The online version of this article (10.1186/s12884-018-1872-4) contains supplementary material, which is available to authorized users.

## Background

Cost-effective health strategies are known to save women’s lives and to protect their newborns [1]. In fact, a significant proportion of newborn and maternal deaths can be prevented through simple and cost-effective strategies [1]. In Mexico, routine delivery practices are not necessarily based on World Health Organization (WHO) recommendations, but rather on convention and out-of-date information [2–4]. Several of these practices are actually potentially harmful to women but are routinely used because of the hierarchical organization of clinical teams in Mexican hospitals [5]. At hospitals in Mexico, medical authorities are generally not questioned, and the implementation of evidence-based practices is lacking [6].

Maternal and newborn care is dependent on effective multiprofessional teams of health care providers. The current challenge for training programs is to increase the capacity and experience of these multidisciplinary teams [7]. Obstetric care in Mexico is provided primarily in health institutions [8], underscoring the importance of improving the quality of care through the appropriate training of health provider teams attending deliveries. The complexity of training these teams requires multifunctional systems that go beyond organizational divisions to facilitate communication, accountability, and the maintenance of supplies and equipment [9].

Many studies on maternal mortality have indicated that the quality of obstetric services, as well as the provision of timely and adequate care for obstetric emergencies, is key in reducing institutional maternal morbidity and mortality [9–12]. Models of traditional training, didactic sessions, and the introduction of guidelines and protocols have failed to show the expected results on relevant indicators and have not yielded an increase in the performance of evidence-based practices [13]. There is an urgent need to update the skills of professionals who do not currently have the competencies required to provide emergency obstetric care as a team [14].

An overview of interventions aimed at improving the performance of health professionals in low-income countries suggests that the simple dissemination of written guidelines is often ineffective, whereas educational outreach visits and audits with feedback are generally effective [14, 15]. Additionally, multifaceted interventions might be more effective than single interventions [14]. A previous impact evaluation showed that PRONTO, an obstetric emergency-management training intervention for health providers based on highly realistic simulation and team training, had a significant effect on several routinely performed obstetric practices in Mexican hospitals. However, an important issue left unexplored by this past work involves the magnitude of the spillover or positive externality of the intervention: Only 20% of the eligible health providers were actually trained, but it was hypothesized that they would transmit their newly acquired knowledge and abilities to their peers, cascading the impact. Moreover, this evaluation also raised new questions about sustainability, because a marked dilution of the effect was observed over the course at one year [16]. This dilution may be explained by the rotation of trained health providers, the abandonment of newly learned practices by trained providers, or a combination of these two factors.

The present study built on the previous report of the average treatment effect (ATE) of PRONTO on routine practices. We sought to deepen the understanding of the mechanisms explaining the dilution of the effect by incorporating newly obtained information and a different analytical strategy. Instead of comparing practices performed in treated vs. untreated hospitals as a whole, we focused on the participants who actually received the treatment (i.e., participated in the training) to estimate the average treatment on the treated (ATT) effect. The ATT estimation allowed us to approximate how large the total ATE would be if a larger proportion of personnel had taken the training. Additionally, by comparing the effect on the trainees to the effect observed on those who did not actually attend the training but there were working side by side who those who did indeed attend (Average Treatment effect on the Non-Treated or ATNT), it can also provide insights on how well the competencies acquired through training are transmitted among peers in health institutions.

Our analysis addressed two key questions left unanswered by the previous ATE analysis of the impact of PRONTO on routine practices during delivery [16]: 1) What is the magnitude of the positive externalities of the intervention in terms of the transmission of knowledge and behavior changes within the treated hospitals among those providers who did not receive the training? and 2) What is the reason for the dilution of the effect of PRONTO on some of the outcomes?

## Methods

### Intervention

The PRONTO simulation-based training, a training course on the management of obstetric emergencies, was developed and piloted in Mexico in 2009. Although the training is not oriented toward the management of normal deliveries, that topic is included in several sessions of the curriculum [17]. A cluster randomized controlled trial was conducted in 24 hospitals in three Mexican states to evaluate the impact of PRONTO. A total of 450 participants (54% physicians and 46% nurses) received the PRONTO training at the 12 intervention hospitals. The selection of these participants was carried out by the authorities in the selected health facilities, but the main selection criterion was that the trainees were personnel who attended deliveries or worked in emergency or delivery rooms [16]. For the original study, hospitals were matched one-to-one based on similar characteristics and incidence of obstetric complications; one member of the hospital pair was then randomly assigned to receive the training. Further details on the original impact evaluation study and the methods used in the training have been previously published [18].

### Data collection: Observation of deliveries

Data collection began in August 2010, and the follow-up concluded in March 2013 in the 24 participating hospitals. Five field workers were instructed to conduct a structured observation of births from the beginning of the second stage of labor until 10–20 min after the third stage was completed, using a paper-based checklist to collect the data. During each data collection period (the baseline and follow-ups at 4, 8, and 12 months after the intervention), the goal was to observe at least 10 vaginal births attended by different providers on different shifts at each hospital over a maximum 5-day observation period. Prior to the observation of births, both the health provider and the laboring woman provided oral consent. The observers were not blinded to the treatment allocation, but they were blinded to the treatment status of individual providers. A more detailed explanation is provided elsewhere [16].

### Retrieval of additional information

The original dataset analyzed by Fritz, et al. (2017) was a set of 641 observed deliveries [16]. However, to estimate the ATT effect, it was necessary to create a unique identifier for each health provider and to distinguish those providers who were trained using PRONTO from those who were not. Because the original study design was not planned to allow for analysis at the provider level, no unique provider identifier was recorded. Rather, the names of delivery attendants were recorded in an informal, non-standardized fashion. Consequently, there was vast heterogeneity in the way these names were written, which made it impossible to create unique identifiers in a straightforward manner. An additional problem was that the gender of the provider was not recorded. In some cases, we were able to infer gender from the name. However, this was not always possible, especially for interns, who were usually referred to in hospital notes only by the prefix MIP and their family name (e.g., “MIP Perez,” which means “Undergraduate Intern Medic Perez” and is a gender-neutral expression in Spanish).

A uniformization of the providers’ names was performed using the Stata command STRGROUP [19], which matches similar names of providers within each hospital and profession. The algorithm for the matching between all pairwise combinations of strings into the “provider name” variable considered the estimation of the Levenshtein distance, defined as the minimum number of single-character edits (i.e., insertions, deletions, or substitutions) required to change one string into the other [20]. A normalization was then performed by dividing the distance by the length of the shorter string in the comparison. Subsequently, we matched a string pair if the names’ normalized distance was less than or equal to a threshold of 0.25, which corresponded to the 10th percentile of the distribution of Levenshtein distances. The algorithm was also applied to identify name similarities among the list of participants in the training, which were stored in a different dataset. Finally, each set of matches was assigned a unique provider identification number, and this was manually re-checked prior to analysis. The health provider’s gender was inferred from their name or from clues such as the use of “Dr.” or “Dra.” as prefixes for the name. We were unable to retrieve information on the name of the provider for 3% of the observed deliveries, and we were unable to identify the gender of the provider for 12% of cases. We were able to ascertain gender for 100% of the treated providers. The study protocol for the original study (Reference 845), as well as for additional data gathering and analysis on individual providers (Reference 733) were reviewed and approved by the Ethics and Research Committees of the National Institute of Public Health in Cuernavaca, Mexico.

### Analytic sample

The original dataset of 641 observed deliveries was collapsed by hospital and stage (4, 8, and 12 months following the training) to a dataset of 310 providers who attended at least one delivery after the time of the training. We summarized the outcomes as the proportion of attended deliveries in which routine practice was followed. The unit of analysis was the i^th^ provider at the t^th^ stage of the study, for a final analytic sample size of 356 observations, of which 50 corresponded to observations of trained providers, 141 were observations of non-trained providers in treated hospitals, and 165 were potential controls (providers observed in control hospitals). The study flow chart is included as supplementary material [see Additional file 1].

### Variable definitions

The main outcomes were the routine practices performed during delivery. Three practices can improve patient outcomes and prevent significant childbirth complications, according to the WHO [9]: 1) Active management of the third stage of labor (AMTSL), defined as (1st Step) applying 10 international units of oxytocin in the first minute after the birth of the baby, (2nd Step) traction and countertraction of the umbilical cord, and (3rd Step) uterine massage immediately after the birth of the placenta; 2) use of delayed cord clamping (DCC), defined as a delay in the clamping of the umbilical cord of at least 60 s after the birth of the baby; and 3) Skin-to-skin contact (SSC), defined as immediate contact or attachment between mother and child after birth. Three unsubstantiated and harmful practices that can worsen patient outcomes if used routinely, according to the WHO [9] are as follows: 1) episiotomy, defined as the performance of a surgical incision in the woman’s perineum, including the skin, muscular plane, and vaginal mucosa; 2) fundal uterine pressure (the Kristeller maneuver), defined as the application of manual pressure on the upper part of the uterus directed toward the birth canal; and 3) uterine sweeping, defined as the introduction of a hand or a gauze-wrapped instrument into the fundus of the uterus after the birth of the placenta.

The main explanatory variable was participation in the PRONTO training (yes/no). Important covariates at the individual level were the health provider’s gender, shift, and profession.

### Statistical analysis

The following equation is the mathematical approach used to estimate the ATT effect of the PRONTO training (*δ*_1_), which can be understood as the expected difference on the performance of routine practices (AMTSL, DCC, SSC, episiotomy, fundal pressure, and uterine sweeping) between those in the intervention group and those who were trained:

1 $$ {\delta}_1=E\left({y}_1-{y}_0\left|t\right.=1\right) $$

In this equation, y_1_ is the probability of a trained provider performing a routine practice and y_0_ is the probability of practice performance with no training (counterfactual), given that the participant was actually trained. The ATT impact estimators were obtained by comparing the prevalence of practices performed by participants with the prevalence among non-participants who would have taken the training had they been offered the opportunity to do so, the prevalence among this non-participants serves as a proxy of the counterfactual: the prevalence on those who were indeed trained in the hypothetical situation were the training never took place. To accomplish this, and because participants in the training were not randomly selected within the treated hospitals, we matched each trained provider with a non-trained provider from the control hospitals, this procedure relies on the assumption that that conditional on the matched covariates, the trained providers and non-trained providers are exchangeable [21]. We performed the matching using one-to-one nearest-neighbor matching in terms of the minimum Mahalanobis distance [22], which was defined using the following covariates: matched hospital pair, state, gender, shift, and profession. Additionally, to control for trends in the probability of performing the practices, we matched exactly on time elapsed since the training (4, 8, or 12 months). Because the accuracy of the estimators depended on the comparability of the treatment groups, we performed a sensitivity analysis using matching to the one, two, and three nearest neighbors with the *tebalance* Stata routine. We chose the one-to-one matching procedure, because it provided more than twice the bias reduction (in terms of the average difference in covariates between the groups) yielded by the use of two or more neighbors.

To estimate the possible effect of PRONTO on those who were not directly trained (positive externality), we performed an estimation of the ATE for non-treated providers (ATNT), matching those who did not attend the training but were working in treated hospitals to those working in control hospitals, otherwise following the same approach described above. Finally, to test for possible decay (linear trend) of the probability of treated providers performing the examined practices in the post-training period, we fitted a provider-level fixed effects regression model with robust standard errors and clustering correction at the hospital level, including time after training as a continuous variable with time units of four months (time elapsed between observation rounds). All analyses were performed with Stata 14.0 (StataCorp LP, College Station, TX, USA). All relevant data is included in anonymized form as a supplementary file [see Additional file 2].

## Results

Descriptive statistics on providers who attended the training, providers who did not attend but who worked in treated hospitals, and providers working in control hospitals are presented in Table 1. The providers who took the training are not representative of the composition of providers attending deliveries, because the training attendees were not randomly selected.

**Table 1:** Descriptive statistics on providers attending deliveries in studied hospitals

Variable	Number	Percent	Number	Percent	Number	Percent
Gender	Treated		Non-Treated		Controls	
Male	29	58.0	66	46.8	83	50.3
Female	21	42.0	61	43.3	61	37.0
missing	0	0.0	14	9.9	21	12.7
Total	50	100	141	100	165	100
Profession						
Interns	5	10.0	48	34.0	75	45.5
Social service practitioners	0	0.0	7	4.9	8	4.8
General Practitioners	33	66.0	55	39.0	32	19.4
Residents	0	0.0	8	5.7	9	5.5
Ob-Gyns	10	20.0	17	12.1	39	23.6
Nurses	2	4.0	5	3.6	2	1.2
missing	0	0.0	1	0.7	0	0.0
Total	50	100	141	100	165	100
Shift						
Morning	16	32.0	53	37.6	50	30.3
Afternoon	25	50.0	57	40.4	84	50.91
Night	8	16.0	28	19.9	29	17.6
missing	1	2.0	3	2.1	2	1.2
Total	50	100	141	100	165	100

Approximately 46% of the health providers who attended deliveries in the control hospitals were interns, whereas interns comprised only 10% of the providers attending the training. Most providers taking the training and attending deliveries were either general practitioners (66%) or obstetricians (20%). The proportion of treated providers who were obstetricians was very similar to the overall proportion of delivery-attending providers made up by obstetricians in the control hospitals. No treated residents attended deliveries, whereas 5.5% of the providers attending deliveries in control hospitals were residents. The proportion of treated providers who were nurses was quite low, but it was higher than the proportion of providers attending deliveries who were nurses in the control hospitals (4 vs. 1.2%).

We found that providers trained by PRONTO showed significant increases in the probability of performing the complete AMTSL (21 percentage point (p.p.) increase) and especially the first and third steps (28 and 29 p.p. increase, respectively), as well as skin-to-skin-contact (26 p.p. increase). We also found a negative and significant effect of the PRONTO training on the probability of performing uterine sweeping (26 p.p. decrease) (Table 2).

**Table 2:** Average treatment effect on treated^a^ for probability of performing routine delivery practices among trained providers

Practice	N^c^	β	95% CI		*p*-value
Complete AMTSL^b^	48	0.21	0.06	0.35	0.005
1st step of AMTSL	48	0.28	0.09	0.46	0.003
2nd step of AMTSL	49	0.08	−0.04	0.20	0.198
3rd step of AMTSL	49	0.29	0.10	0.48	0.003
Skin-to-skin contact	49	0.26	0.14	0.38	< 0.001
Delayed cord clamping	24	0.21	−0.07	0.49	0.148
Episiotomy	49	−0.06	−0.27	0.16	0.592
Fundal pressure (Kristeller maneuver)	49	0.10	−0.04	0.23	0.157
Uterine sweeping	48	−0.26	−0.44	−0.08	0.004

^a^Impact estimates were obtained by Mahalanobis distance nearest-neighbor matching in terms of the following covariates: matched hospital pair, state, gender, work shift, and profession, with exact matching on time elapsed since the training

^b^AMTSL: active management of the third stage of labor

^c^Number of trained providers

**Fig. 1:**
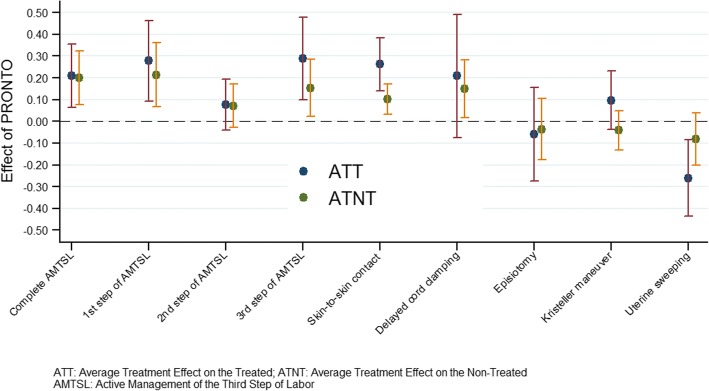
Average treatment effects for probability of performing routine delivery practices among providers in treated hospitals

We found that non-trained providers working in treated hospitals (Table 3) also showed marked and significant changes in the same practices, except for uterine sweeping, and there was a significant impact on the performance of delayed cord clamping (15 p.p.). No significant changes in the performance of episiotomy or the Kristeller maneuver were found in any of the models. As expected, the effect on the treated providers was substantially larger, on average, than the effect on non-treated providers working in treated hospitals (Fig. 1).

**Table 3:** Average treatment effect on non-treated^a^ for probability of performing routine delivery practices among untrained providers

Practice	N^c^	β	95% CI		*p*-value
Complete AMTSL^b^	118	0.20	0.08	0.32	0.001
1st step of AMTSL	124	0.21	0.07	0.36	0.005
2nd step of AMTSL	123	0.07	−0.03	0.17	0.160
3rd step of AMTSL	118	0.15	0.02	0.28	0.021
Skin-to-skin contact	124	0.10	0.03	0.17	0.004
Delayed cord clamping	50	0.15	0.02	0.28	0.027
Episiotomy	123	−0.04	−0.18	0.10	0.611
Fundal pressure (Kristeller maneuver)	123	−0.04	−0.13	0.05	0.363
Uterine sweeping	123	−0.08	−0.20	0.04	0.184

^a^Impact estimates were obtained by Mahalanobis distance nearest-neighbor matching in terms of the following covariates: matched hospital pair, state, gender, work shift, and profession, with exact matching on time elapsed since the training

^b^AMTSL: active management of the third stage of labor

^c^Number of untrained providers working in treated hospitals

Finally, we did not find evidence of a significant decay of the probability of performing any of the routine practices (or of an increase in the performance of harmful practices) over time among the treated providers. Most time trend coefficients indicated very close to zero change, except for two practices: uterine sweeping and 1st step of AMTSL, in which changes (non-significant) occurred in the expected direction (negative and positive respectively) (Table 4).

**Table 4:** Linear time trend of probability^a^ of performing routine delivery practices among trained providers

Practice	N^c^	β^a^	95% CI		*p*-value
Complete AMTSL^b^	48	0.03	− 0.18	0.24	0.768
1st step of AMTSL	48	0.15	−0.28	0.57	0.462
2nd step of AMTSL	49	0.00	(non estimable)		
3rd step of AMTSL	49	−0.09	−0.42	0.24	0.571
Skin-to-skin contact	49	0.00	(non estimable)		
Delayed cord clamping	24	−0.03	−0.33	0.27	0.835
Episiotomy	49	0.03	−0.36	0.42	0.872
Fundal pressure (Kristeller maneuver)	49	−0.06	−0.20	0.08	0.384
Uterine sweeping	48	−0.21	−0.47	0.06	0.120

^a^Fixed effects linear model with robust standard errors and clustering correction at the hospital level. □ coefficients are changes in probability per 4 elapsed months

^b^AMTSL: active management of the third stage of labor

^c^Number of trained providers working in treated hospitals

## Discussion

In this study, we used a matching procedure to estimate the ATT impact of receiving the intervention (PRONTO training) on the probability of performing routine practices. This approach may allow us to estimate the effect of the intervention if all providers at the trained facilities had attended the program. In a previous impact evaluation of PRONTO, significant changes in the probability of routine practices being performed were found, but it was not known if these were diluted because of the relatively small fraction of eligible health providers who were trained or to what extent there was a spillover effect [16]. Our analysis concludes that the spillover effect is about 53% as large, on average, as the effect on the treated providers.

That no effect was observed for several of the practices, namely episiotomy and fundal pressure, reflects how deeply engrained these practices are in the culture of care and underscores the importance of interventions designed to impact the use of these practices earlier in the providers’ careers, even during medical school. The results also suggest that the previously observed dilution of the effect of the intervention might be more related to the rotation of personnel than to providers returning to their old habits. This suggestion is supported by more than 40% of deliveries being attended by interns, who only spend 2–4 months on the obstetrics rotation.

Several previous studies have estimated the treatment effects of a program evaluation approach associated with maternal health outcomes. Kaul et al. used propensity score matching to estimate ATT associated with deliveries at health care institutions for women in India [23]. Habibov et al. used probit models to estimate the effects of delivering in health care facilities on the probability of child survival, taking into account self-selection into the treatment, with nonrandomized data from a cross-sectional survey in Azerbaijan [24]. Wang et al. used propensity score matching to estimate the impact of health insurance status on the use of antenatal care and facility-based delivery care in eight countries of sub-Saharan Africa, West Asia, and South and Southeast Asia [25].

Renfrew and colleagues established a comprehensive list of beneficial and harmful practices during intrapartum care [26]. Included in this list are those practices that can be easily addressed within the context of a simulation training program, such as PRONTO, with the primary focus of improving obstetric and neonatal emergency care. Harmful delivery practices in Mexico are likely contributing to the country’s high rate of caesarean sections [2–4]. This may be because of a tendency to practice traditional authority-based medicine instead of applying new evidence-based practices [27].

A group of researchers who conducted a study in Latin America reported that, within institutions, interventions founded in evidence-based medicine were generally underused, while ineffective or even harmful practices continue to be used. This study showed that the intervention, carried out within a randomized trial and relying on a combination of practice and knowledge was significant in terms of changing delivery practices in favor of those based on evidence [15].

Several study limitations related to our analysis must be acknowledged. As the study was not originally designed to examine providers, we were only able to match on a limited number of available covariates. Data on provider gender was missing for a sizeable fraction of the original sample of observed deliveries. We were unable to identify gender in 12% of the original sample, although most of these providers were interns, who constituted only a small proportion of the treated providers. Gender was identified for 100% of the trained sample. In addition, the sample size of providers who actually received the training was quite small, which reduced the power of the study. Nonetheless, this sample size was large enough for us to detect significant effects for most of the indicators examined.

## Conclusions

The analysis of the impact of PRONTO training on providers yields new insights on the true efficacy of this approach and, through comparing the estimated effect on the treated vs. that on the non-treated providers, the extent to which knowledge and abilities acquired through this training are disseminated within hospitals. It also further highlights the importance of continuous, ongoing training to counter the dilution effect, which is probably caused by the rotation of personnel within health institutions in Mexico, to ensure the provision of quality maternal and newborn care.

## Additional files

Additional file 1: Study Flow Chart. The process from hospital-level randomization leading to the provider-level analytic sample is described in this chart. (DOC 41 kb)

Additional file 2: Provider-level dataset. This dataset includes all relevant anonymized information of the study participants’ as well as the birth observation variables during baseline and follow ups. (XLS 169 kb)

## Abbreviations

AMTSL: active management of the third stage of labor
ATE: average treatment effect
ATT: average treatment on the treated
DCC: delayed cord clamping
p.p.: percentage points
PRONTO: Programa de Rescate Obstétrico y Neonatal: Tratamiento Óptimo y Oportuno
SSC: skin-to-skin contact
WHO: World Health Organization
