# Supplementary material for: Health providers pass knowledge and abilities acquired by training in obstetric emergencies to their peers: the average treatment on the treated effect of PRONTO on delivery attendance in Mexico
Source: BMC Pregnancy Childbirth. 2018 Jun 15;18:232. doi: 10.1186/s12884-018-1872-4 (PMC6003075; doi:10.1186/s12884-018-1872-4)
Supplement: Supplementary file 1 — Study Flow Chart. The process from hospital-level randomization leading to the provider-level analytic sample is described in this chart. (DOC 41 kb) [file 12884_2018_1872_MOESM1_ESM.doc]

**Study Flow Chart**

**Allocation**

**ATE Analysis**

**Follow-Up**

**Enrollment**

**ATT Analysis**

Mexican Public Hospitals Assessed for eligibility (n=570)

Excluded (n=546)

♦  Not meeting inclusion criteria (n=486)

♦  Not high on mortality list (n=60)

Analysed (n=12 hospitals, 318 observed deliveries, 191 providers)
♦ Excluded from analysis (n=0)

Lost to follow-up (n=0)

Discontinued intervention (n=0)

Allocated to intervention (training) (n=12)

♦ Received allocated intervention (n=7 )

♦ Replaced due to reconstruction, re-structuring or reorganization; re-matched and randomized (n=5 )

Lost to follow-up (n=0)

Discontinued intervention (n=0)

Allocated to intervention (control) (n=12)

♦ Received allocated intervention (n=6)

♦ Replaced due to reconstruction, re-structuring or reorganization; re-matched and randomized (n=6)

Analysed (n=12 hospitals, 323 observed deliveries, 165 providers )
♦ Excluded from analysis (n=0)

Randomized (n=24)

Matched and Analysed (n=50 trained providers and n=127 non trained providers)

♦ Excluded from analysis due to missing values in covariates (n=14)

Matched and Analysed (n=144 non trained providers)

♦ Excluded from analysis due to missing values in covariates (n=21)
